# Supplementary material for: Sodium Alginate-Based MgO Nanoparticles Coupled Antibiotics as Safe and Effective Antimicrobial Candidates against Staphylococcus aureus of Houbara Bustard Birds
Source: Biomedicines. 2023 Jul 11;11(7):1959. doi: 10.3390/biomedicines11071959 (PMC10377686; doi:10.3390/biomedicines11071959)
Supplement: Supplementary file 1 [file biomedicines-11-01959-s001.zip › biomedicines-2431592-supplementary.pdf]

Table S1. Summary of structural parameters obtained from XRD data.

**Parameters**	**Results**
ICDD card #	77–2364
Crystal system	Cubic
Space group	Fm-3 m
Space group no.	225
No. of atoms in unit cell	27
**Cell parameters**	
a	3.98300Å
**Atom coordinates**	
x, y and z coordinates of Magnesium	0.00, 0.00, 0.00
x, y and z coordinates of Oxygen	0.50, 0.50, 0.50
**Density**	4.23700 g/cm^3
**d spacing and hkl**	2.299 and (111)
	1.9915 and (200)
	1.408 and (220)
	1.2009 and (311)
	1.1498 and (222)

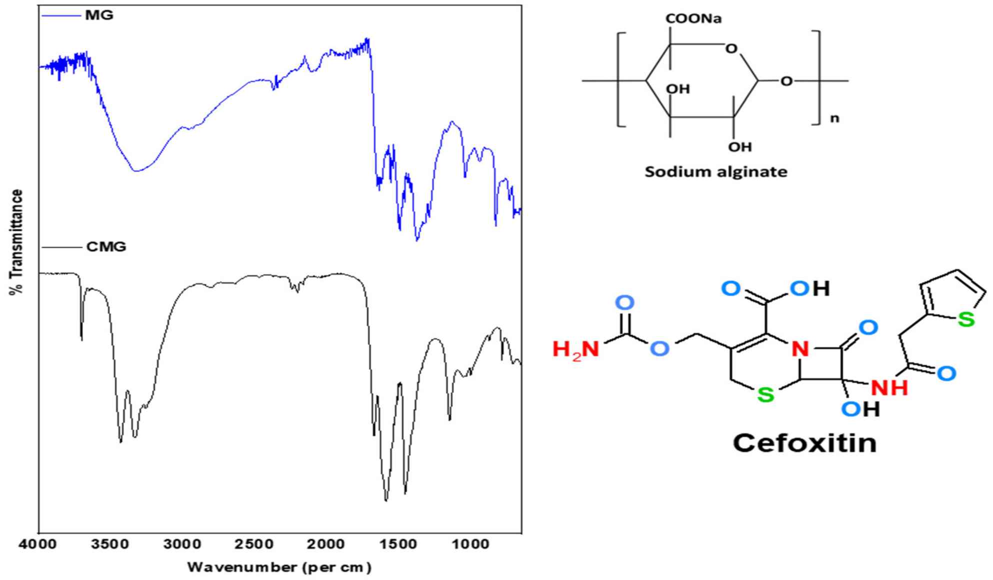

Figure S1. FTIR pattern of MG and GMC.

In Figure S1, strong bands are observed at 1500–1600 cm^{-1} representing presence of carbonyl functional group in CMG. Due to presence of amine (NH_2 and NH) groups, two peaks are observed around 3300–3600 cm^{-1}. In case of MG, amine groups are absent. Only hydroxyl groups are present. So a broad band is observed around 3300–3600 cm^{-1}. MgO peaks are present before 1000 cm^{-1}. Comparison of both patterns confirm that drug has been coated because characteristic peaks of amine are obtained.

Table S2. Risk factor analysis of *S. aureus* isolated from enteric source of Houbara bustard birds.

Variable	Levels	Screened	Positive	Prevalence (%)	*p*-value	CI 95%	
						Lower ratio	Upper ratio
Gender	Male	35	18	51.42	0.579	35.57	67.01
	Female	70	40	57.14		45.48	68.06
Age	Up to 6 months	15	8	61.66	0.427	30.11	75.19
	7–12 month	36	23	63.88		47.58	77.53
	Above 1 years	54	27	50		37.11	62.89
Housing system	Open housing	67	33	49.25	0.102	37.65	60.93
	Pen	38	25	65.78		49.89	78.79
Feeding system	Poultry feed	38	11	28.95	<0.01	17.01	44.76
	Poultry feed plus Scavenger	67	47	70.15		58.35	79.77
Season	Spring	40	25	6.25	0.114	47.03	75.78
	Winter	30	15	50		33.15	66.85
	Summer	35	18	51.42		35.57	67.01
Gastro intestinal Parasites	Yes	44	38	86.36	<0.01	73.29	93.59
	No	61	20	32.78		22.34	45.28
Exposure of antibiotics	Frequent	25	21	84	<0.01	65.35	93.6
	Occasional	30	22	73.33		55.55	85.81
	No use	50	15	30		19.1	43.75
Type of antibiotic used	Beta-lactam	50	39	78	<0.01	19.1	43.75
	Other	10	4	40		16.82	68.73
	NA	50	15	30		19.1	43.75

$p < 0.05$ indicate significant association, MMS = Methyl methanesulphonate.

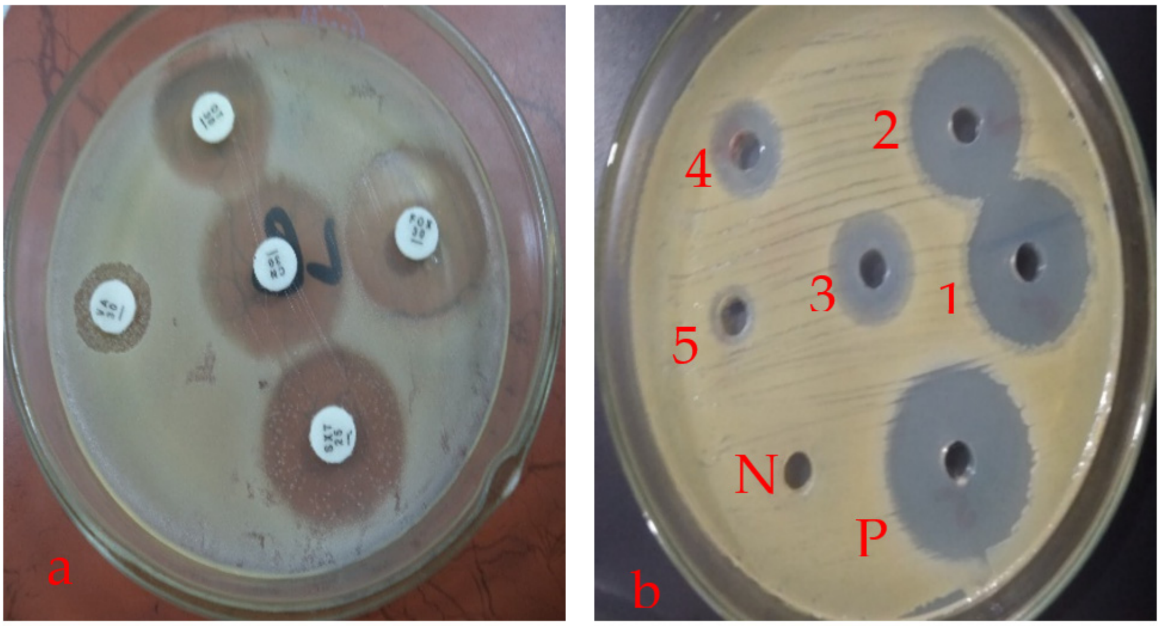

Figure S2. Antibacterial activity by disc diffusion and well diffusion method (**a**) for antibiotic susceptibility and well diffusion method (**b**) for nanocomposites against S. aureus. *n* = negative control, P = positive control, 1–5 = nanocomposite preparations.
